# Development of 3D Printed Enzymatic Microreactors for Lipase-Catalyzed Reactions in Deep Eutectic Solvent-Based Media

**DOI:** 10.3390/mi13111954

**Published:** 2022-11-11

**Authors:** Myrto G. Bellou, Elena Gkantzou, Anastasia Skonta, Dimitrios Moschovas, Konstantinos Spyrou, Apostolos Avgeropoulos, Dimitrios Gournis, Haralambos Stamatis

**Affiliations:** 1 Laboratory of Biotechnology, Department of Biological Applications and Technologies, University of Ioannina, 45110 Ioannina, Greece; 2 Department of Materials Science and Engineering, University of Ioannina, 45110 Ioannina, Greece

**Keywords:** 3D printing, immobilization, enzyme microreactor, deep eutectic solvents, lipase

## Abstract

In this study, 3D printing technology was exploited for the development of immobilized enzyme microreactors that could be used for biocatalytic processes in Deep Eutectic Solvent (DES)-based media. 3D-printed polylactic acid (PLA) microwell plates or tubular microfluidic reactors were modified with polyethylenimine (PEI) and lipase from *Candida antarctica* (CALB) was covalently immobilized in the interior of each structure. DESs were found to have a negligible effect on the activity and stability of CALB, and the system proved highly stable and reusable in the presence of DESs for the hydrolysis of p-nitrophenyl butyrate (p-NPB). A kinetic study under flow conditions revealed an enhancement of substrate accessibility in the presence of Betaine: Glycerol (Bet:Gly) DES, while the system was not severely affected by diffusion limitations. Incubation of microreactors in 100% Bet:Gly preserved the enzyme activity by 53% for 30 days of storage at 60 °C, while the buffer-stored sample had already been deactivated. The microfluidic enzyme reactor was efficiently used for the trans-esterification of ethyl ferulate (EF) with glycerol towards the production of glyceryl ferulate (GF), known for its antioxidant potential. The biocatalytic process under continuous flow conditions exhibited 23 times higher productivity than the batch reaction system. This study featured an effective and robust biocatalytic system with immobilized lipase that can be used both in hydrolytic and synthetic applications, while further optimization is expected to upgrade the microreactor system performance.

## 1. Introduction

The narrative about miniaturized analytical systems (MAS) has been rapidly established in recent years, and, currently, 3D printing technology is gaining more and more space in this field of study. It is commonly accepted that microfluidic networks have greatly impacted the way experimentation and data analysis is performed on several levels, from complexity to robustness and testing speed. Incorporating additive manufacturing into this kind of microsystem allows the creation of integrated devices by end-users and at the point of use while providing freedom to create and change without prior knowledge [1]. Three-dimensional printed microfluidic channels have already been demonstrated for bio-based applications like protein detection, cell deposition and simulation, bacterial communities, tissue constructs, organ-on-a-chip, and microfluidic reactionware [2,3].

Enzymes are a class of macromolecule consisting of one or more folded amino chains forming a three-dimensional structure. They have the ability to bind molecules in the substrate binding pocket and perform catalysis in the adjacent active site, and they are especially known for their ability to surpass other catalysts with respect to chemical specificity, and regio- and enantio-selectivity. Biocatalysis, as a research field, employs one or more enzymes (biocatalysts) to convert substrates into higher-value compounds, and biocatalytic systems have been realized in different industrial processes, from food products, pharmaceuticals, cosmetics, and textiles, to biofuels and water purification [4]. However, the water solubility of biocatalysts hinders their recovery and recyclability, essential prerequisites for a circular economy. The development of enzyme immobilization has proved a revolutionary technology in this respect, changing radically enzyme performance, since biocatalytic systems that are highly reusable and stable under different reaction conditions are now a fact. Different types of biocatalytic systems with immobilized enzymes have been explored, utilizing nanomaterials or micro-scale materials as immobilization scaffolds [5,6]. Three-dimensional printing technology has come to offer such immobilization scaffolds, which can be designed with programmable structures in a scalable and cost-effective form. The advantages of merging these technologies include strong operability since complex structures are printed with pores and channels at low cost, while the enzyme-immobilized scaffold will not suffer material loss or make use of undesirable chemical reagents [7]. In addition, 3D printing materials are (bio)degradable and recyclable, leaving no environmental footprint. In view of these advantages, researchers have explored 3D printing for enzyme immobilization for industrial and healthcare applications [8].

Deep Eutectic Solvents (DESs) and their natural counterparts (NaDESs) have lately been employed in the biocatalysis field, where a plethora of different enzymes (mainly hydrolases and oxidoreductases), such as β-glucosidases [9,10], cytochrome P450 [11], laccases [12,13], and lipases [14], have been studied in the presence of DESs that act as co-solvents in DES-buffer solutions. This is because they are nontoxic, nonvolatile, nonflammable, and even biodegradable, rendering them a cost-efficient and enzyme-compatible reaction media [15]. DESs have a eutectic nature and are prepared by mixing a hydrogen bond donor (HBD) and a hydrogen bond acceptor (HBA) while being stirred and heated until a liquid is formed. The DES retains its liquid form at ambient temperature due to the formation of intramolecular hydrogen bonds, thus leading to a decreased freezing point. DESs are usually produced by using inexpensive quaternary ammonium salts such as choline chloride, millions of tons of which are produced every year, combined with HBDs from renewable sources such as sugars, amino acids, alcohols, and process wastes such as glycerol, depending on the desired application [16]. In addition to the concept of reaction media, research is moving toward the 2-in-1 concept in which the DES simultaneously acts as the reaction medium and the substrate pool, one (or both) of its components being the substrate of the biotransformation reaction, thus promoting the sustainability of the system [16,17,18,19,20].

Combining the above knowledge, this study proposes the use of 3D printing technology to develop microdevices for biocatalytic applications exploiting the effect of DESs on immobilized enzyme performance. The material of choice was polylactic acid (PLA), a widely used thermoplastic polymer known for its excellent biocompatibility and biodegradability, exploited in several bio-related applications as a common 3D printable material [21]. Our group has already demonstrated the potential of PLA for immobilized enzyme microreactor development [22]. However, biocatalytic processes in 3D-printed scaffolds are currently being performed merely in aqueous solution [23], since organic solvents that have been exploited for synthetic reactions with other support materials [24] have been proved improper for 3D-printed PLA structures, as was indicated in a recent study [25]. For this reason, herein, DESs were explored as alternative solvents for biocatalytic processes both in aqueous and non-aqueous reaction systems. As a biocatalyst, lipase from *Candida antarctica* B was used, one of the first enzymes exploited in DES-based processes and widely studied for its diverse biotechnological applications [26]. The incorporation of DESs into continuous biocatalytic processes in microreactor systems has been recently exerted by experts in the field of the application of DESs in biocatalysis [18,27]. More specifically, it has been proposed that the employment of DESs as alternative solvents in microfluidic bioprocesses renders these systems sustainable and highly efficient [27].

Τhis study describes the use of 3D-printed well plates as screening platforms for immobilized lipase performance in the presence of different DESs. The most promising DES in terms of enzyme activity and stability was tested for its performance as a reaction medium component for continuous flow biocatalysis in 3D-printed microreactors. Biocatalytic characteristics for immobilized lipase were investigated in terms of enzyme concentration, flow rate (kinetic characteristics), operational stability, and stability after storage in 100% DES solution. The potential for a lipase-catalyzed transesterification reaction in a 100% DES medium was also demonstrated in a continuous flow system.

## 2. Materials and Methods

Choline chloride > 98%, betaine 98%, D(+)-glucose 99.5%, D(-)-fructose, polyethyleneimine, branched (PEI), ethyl-4-hydroxy-3-methoxycinnamate (ethyl ferulate) 4-nitrophenyl butyrate (pNPB) > 98%, and p-nitrophenol (pNP) were obtained from Sigma-Aldrich, St. Louis, MI, USA. Choline dihydrogen phosphate >98% was bought from IOLITEC, (Heilbronn, Germany), ethylammonium chloride from Merck (Sigma-Aldrich, USA), urea > 99% from Fluka (Sigma-Aldrich, USA), glycerol 0.5% max water, and Glutaraldehyde 25% solution from Fisher Scientific (Hampton, NH, USA), ethylene glycol from AppliChem (Darmstadt, Germany), 1,2-butanediol > 98% from TCI (Japan), 1,2-propanediol 99% from Acros Organics (Waltham, MA, USA), and sodium hydroxide > 98% from Panreac (Barcelona, Spain). The HPLC solvents acetonitrile, methanol, and water were of HPLC grade and the water used was double distilled. The lipases from *Candida antarctica* Lipozyme CALB L (liquid) and Novozym 435 (immobilized) were kindly offered by Novozymes (Bagsværd, Denmark). PLA filament designed for use with FDM 3D printers (PrimaValue™) was purchased from Prima Creator (Malmö, Sweden). The 3D printer used was an Ender 5 from Creality 3D (Shenzhen, China).

### 2.1. Three-Dimensional Design and Printing of PLA Models

**Figure 1:**
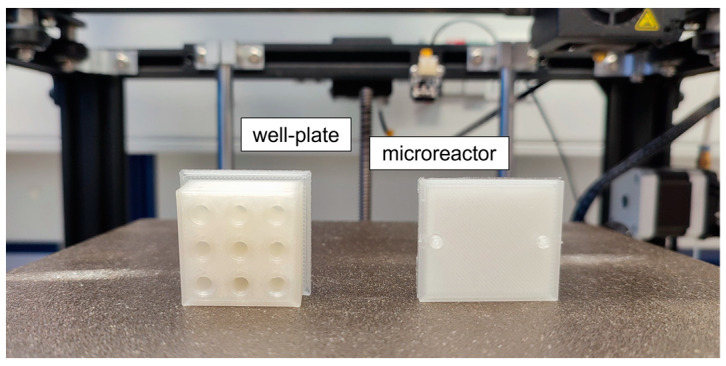
3D-printed models made of natural polylactic acid (PLA). The well plate can be designed with a customized number of wells depending on the experimental needs, while the microreactor bares a serpentine-like internal shape in order to hold more volume in a smaller space. The models are ready-to-use after printing (no post-printing treatment needed).

PLA models were designed using the Fusion 360 CAD program from Autodesk (California, USA), as described in our previous works [22,28]. Briefly, well plates with a customized number of wells were designed with a 150 μL volume capacity for each well. Accordingly, microreactors with a serpentine-like internal structure were designed with a 150 μL working volume. Examples of a nine-well plate and a microreactor model are presented in Figure 1. The final designs were forwarded to the slicing software Cura (Ultimaker, Utrecht, The Netherlands), where appropriate slicing settings were applied, depending on the filament type and the 3D printer used. The printing parameters set by the slicing software are summarized in Table 1. The final printed models were leak-free and ready to use for further experimentation. This was confirmed by filling the printed models with buffer solution and leaving them for 24 h at room temperature. At the end of this period, the total volume collected was the same as the total volume loaded, so no leakage was observed. Additionally, when different flow rates were applied in the microreactor, the total volumes measured at the input and output of the microreactor were identical.

### 2.2. Surface Modification and Enzyme Immobilization on PLA Models

The modification of the PLA scaffolds and the immobilization of lipase were based on previously reported 3D-printed PLA modification protocol [29]. Sodium hydroxide (NaOH) was used for surface etching in order to promote hydrophilicity of the PLA surface, and polyethylenimine (PEI) was used as the main modification agent. Previous studies have shown that NaOH treatment significantly enhances PLA surface roughness [30], making interactions with modification agents more feasible [31,32]. Covalent and non-covalent immobilization methods were investigated. Lipase was either linked on the PEI surface by a glutaraldehyde cross-linking method or absorbed on the PEI-modified PLA, and the corresponding enzyme activity was determined. The results are presented in Supplementary Figure S2, and the covalent linkage was chosen for the subsequent studies.

Firstly, the PLA surface was modified with sodium hydroxide (NaOH). A 150 μL sample of 1 M aqueous solution was added to each well followed by incubation at 25 °C for 2 h. Afterward, the NaOH solution was removed, and the wells were washed twice using 150 μL of double distilled water. Then, the well plate was dried under vacuum for 30 min. Next, 150 μL of 2 mg/mL PEI aqueous solution were added to each well and incubated at 25 °C for 1h. After the incubation, the PEI solution was removed, and the wells were washed twice using 150 μL of double-distilled water. Then, the plate was dried under a N_2_ stream for 30 s. In the next step, glutaraldehyde (GA) was used as the cross-linking agent between PEI and Candida antarctica lipase B (CALB) for the covalent immobilization of the enzyme on the support. A 150 μL sample of 2.5% *v*/*v* GA solution in 50 mM pH 7 phosphate buffer was added to each well and incubation at 30 °C for 1 h followed. After, the GA solution was removed, the wells were washed thrice using 150 μL of 50 mM pH 7 phosphate buffer. The well plate was dried under a N_2_ stream for 30 s. For the enzyme immobilization, a solution of 0.175 mg/mL CALB was prepared in 50 mM phosphate buffer pH 7.5. Each well was filled with 150 μL of the CALB solution and incubation at 30 °C for 1 h followed. Next, the CALB solution was removed and the well was washed thrice using 150 μL of 50 mM phosphate buffer pH 7.5. The well plate was dried under vacuum for 30 min and stored at 4 °C until further use.

The same protocol was used for the modification of the PLA microreactors, with the exception that each microreactor was filled with 150 μL of each solution using disposable 1 mL syringes. The concentrations of the solutions were the same, except for the enzyme solution whose concentration was 0.044 mg/mL and was prepared in 50 mM phosphate buffer pH 7.5 (unless stated otherwise). Additionally, drying was performed under a N_2_ stream for 30 s in every case. The estimation of the concentration of the protein contained in the commercial liquid enzyme preparation was conducted using the Bradford assay [33].

### 2.3. Activity of Immobilized CALB on PLA Well plates

The activity measurement of the immobilized lipase was achieved by photometrically observing the hydrolysis of the model substrate p-nitrophenyl butyrate (pNPB). For this purpose, a stock solution of 12.5 mM pNPB was prepared in acetonitrile. A 150 μL sample of 0.25 mM pNPB solution in 50 mM phosphate buffer pH 7.5 (2% acetonitrile) was added to each well and the well plate was incubated at 40 °C for 5 min in order for the reaction to progress. The absorbance of the p-nitrophenol (pNP) that was produced from the hydrolysis of pNPB was measured in an ELISA plate reader at 405 nm. Measurements were conducted in triplicate. A standard curve of pNP was prepared and used for the calculation of the units of lipase activity.

### 2.4. Preparation of Deep Eutectic Solvents (DESs)

Certain quantities of a series of HBAs and HBDs were weighted and mixed in a glass vial in order to form the DESs of the required molar ratio (Table 2). The prepared mixtures were then incubated at 80 °C at 180 rpm until a clear solution was formed (~1 h). The prepared DESs were stored at 30 °C until further use.

### 2.5. Effect of DESs on the Activity of Immobilized CALB

The activity of the immobilized lipase on PLA wells was screened in a series of DES-buffer solutions of varied DES concentrations. The enzyme activity was measured as described in Section 2.3, with the alteration that the 150 μL of 0.25 mM pNPB solution in 50 mM phosphate buffer pH 7.5 that were added to the wells contained 10% *v*/*v*, 20% or 50% *v*/*v* of each DES. Measurements were conducted in triplicate.

### 2.6. Effect of DESs on the Stability of Immobilized CALB

The stability of the immobilized lipase on PLA well plates was assessed for a 1 h incubation at 40 °C, in the presence of several DESs at different concentrations. First, the enzyme activity was measured as described in Section 2.3. Then, wells were washed thrice with phosphate buffer and dried under a N_2_ stream for 30 s. Then, 150 μL of different concentrations of DESs in 50 mM phosphate buffer pH 7.5 (0, 10, 20, 50, and 100% *v*/*v* DES) were added to the wells, and these were incubated at 40 °C for 1 h. After the incubation, the solutions were removed, and the well plates were washed thrice with phosphate buffer and dried under a N_2_ stream for 30 s. Then, the enzyme activity was measured again. All measurements were conducted in triplicate. For each well, the remaining activity of the lipase is expressed as follows:

Remaining activity (%) = Activity after incubation × 100/Activity before incubation (%)(1)

### 2.7. Storage Stability of Immobilized CALB on PLA Well plates

The storage stability of the immobilized system was evaluated for up to 2 months of storage at 4 °C. The enzyme activity was determined as described in Section 2.3 and wells were washed thrice with phosphate buffer and dried under a N_2_ stream for 30 s. Subsequently, the enzymatic activity of the immobilized CALB was measured after 1, 3, 4, 5, and 8 weeks of storage, and the remaining activity was estimated. Measurements were conducted in triplicate.

### 2.8. Spectroscopic and Morphological Characterization

X-ray photoelectron spectroscopy (XPS) measurements were performed in an ultrahigh vacuum at a base pressure of 7 × 10^−9^ mbar with a SPECS GmbH spectrometer equipped with a monochromatic MgKa source (hv = 1253.6 eV) and a Phoibos-100 hemispherical analyzer (Berlin, Germany). The spectra were collected in normal emission and the energy resolution was set to 1.16 eV to minimize measuring time. The spectral analysis, including a Shirley background subtraction and a peak deconvolution employing mixed Gaussian-Lorentzian functions, was performed in a least squares curve-fitting program (WinSpec) developed at the Laboratoire Interdisciplinaire de Spectroscopie Electronique, University of Namur, Namur, Belgium.

Scanning electron microscopy (SEM) images were obtained using a JEOL JSM-6510 LV SEM Microscope (JEOL Ltd., Tokyo, Japan) equipped with an X–Act EDS-detector by Oxford Instruments, Abingdon, Oxfordshire, UK (an acceleration voltage of 20 kV was applied). Prior to SEM analysis, the samples were coated with an Au/Pd thin film (4–8 nm) in a sputtering machine (SC7620, Quorum Technologies, Lewes, UK).

### 2.9. Assays in PLA Microreactors

#### 2.9.1. Activity Measurement of Immobilized CALB in PLA Microreactors

The activity of the immobilized lipase in the PLA microreactor was determined as described in Section 2.3 with the exception that the substrate solution was loaded in the microreactor by using a syringe pump system with a flow rate of 300 μL/min and residence time (*t*_R_) 30 s (unless stated otherwise). The effluent (150 μL) of the microreactor was collected and the absorbance of the produced pNP was measured in an ELISA plate reader at 405 nm. Measurements were conducted in triplicate.

#### 2.9.2. Effect of Enzyme Concentration

In order to examine the effect of different enzyme concentrations on the activity of the biocatalytic microreactor system, seven enzyme concentrations from 0.01 mg/mL to 3.5 mg/mL were tested. The enzyme immobilization process was performed as described in Section 2.2, except for the varying concentrations of the enzymatic solutions that were used. After the lipase immobilization, the determination of the enzymatic activity of the microreactor followed, as referred at Section 2.9.1. All measurements were conducted in triplicate.

#### 2.9.3. Determination of the Immobilization Yield

The estimation of the immobilization yield was performed as described by the following equation:

Immobilization yield (%) = 100 − (activity after immobilization × 100)/(activity before immobilization) (%)(2)

Measurements were conducted in triplicate.

#### 2.9.4. Effect of Flow Rate in the Presence or Absence of DES

For the evaluation of the effect of the flow rate on the system performance, different substrate concentrations in combination with different flow rates were applied. Substrate solutions containing different concentrations of pNPB (0.125, 0.25, 0.5, 1, 2, and 4 mM) and 10% *v*/*v* DES (in the case of the presence of DES) in 50 mM phosphate buffer pH 7.5 were introduced into the microreactor at four different flow rates (75, 150, 300, and 600 μL/min), respectively. Then, the effluent (150 μL) was collected and the absorbance of the produced pNP was measured in an ELISA plate reader at 405 nm. The Lilly–Hornby model, as an adaptation of the classical Michaelis–Menten kinetic model, is used for investigating the flow kinetics of enzymes immobilized in microreactors and is described by the following equation:

(3) $$f\times {\left[A\right]}_{0}=K_{m\left(app\right)}\times \ln\left(1-f\right)+\frac{C}{Q}$$

where f is the fraction of the substrate converted to the final product during the reaction, Q is the flow rate of the substrate, ${\left[A\right]}_{0}\;$is the initial substrate concentration, ***C*** is the reaction capacity of the microreactor, and $K_{m\left(app\right)}$ is the apparent Michaelis constant.

For the estimation of the lipase kinetics in a batch system, a 5 μL/mL (87.5 μg/mL) stock of free CALB was prepared in 50 mM phosphate buffer pH 7.5. A 3 μL sample of the enzyme stock (1.3 μg/mL) was added to solutions containing different concentrations of pNPB (0.125, 0.25, 0.5, 1, 2, and 4 mM,) and 10% *v*/*v* DES (in the case of the presence of DES) at a final volume of 200 μL in a 96-well plate. The reaction progress was determined spectrophotometrically using an ELISA plate reader at 405 nm, at 40 °C for 5 min, to determine the initial reaction rate. In order to calculate the kinetic constants, nonlinear regression fitting of the initial reaction data depended on the pNPB concentrations was performed, by applying the Michaelis–Menten model (EnzFitter, Biosoft, UK). All measurements were conducted in triplicate.

#### 2.9.5. Operational Stability in the Presence or Absence of DES

The operational stability of the immobilized CALB in PLA microreactors was evaluated for 100 subsequent cycles of use (55 min of continuous use) in the presence or absence of DES. One cycle was defined as the transition of 150 μL of the substrate solution through the microreactor. A syringe pump was used which was loaded with the corresponding volume of 0.25 mM pNPB solution in 50 mM phosphate buffer pH 7.5. After every 10 cycles of use (cycles of 30 s, flow rate 300 μL/min) the tenth effluent (150 μL) was collected, and the absorbance of the produced pNP was measured in an ELISA plate reader at 405 nm. The same procedure was followed for the determination of the operational stability of immobilized lipase in 10% *v*/*v* DES. All measurements were conducted in triplicate.

#### 2.9.6. Thermal Stability of Immobilized CALB in 100% DES

The stability of the immobilized lipase in PLA microreactors was tested in the presence of 100% DES and 100% buffer solution at 60 °C for a total period of one month. Initially, the activity of the lipase was measured as mentioned in Section 2.9.1. Then, the microreactors were washed thrice with 200 μL 50 mM phosphate buffer pH 7.5 and dried under a N_2_ stream for 30 s. Next, the microreactors were filled with 100% DES or 100% phosphate buffer, and the edges were tightly covered with parafilm, in order for the buffer not to evaporate. The microreactors were incubated at 60 °C and at regular time intervals (1, 2, 3, 6, 9, 20, and 30 days), the DES or the buffer was removed, the microreactors were washed and dried, and the remaining enzyme activity was determined as described above. All measurements were conducted in triplicate.

#### 2.9.7. Transesterification Reactions in Microreactor and Batch Systems

The microreactor was used for the transesterification of ethyl ferulate with glycerol. A solution of 2 mM of ethyl ferulate (0.44 mg/mL) in 100% *v*/*v* DES Bet:Gly was prepared and incubated at 60 °C, 800 rpm until ethyl ferulate was fully dissolved in the DES. A peristaltic pump was used for the transition of 150 μL of the substrate solution through the microreactor. The continuous flow transesterification was performed for a total residence time (*t*_R_) of 4 h under a peristaltic flow pattern (24 runs × 10 min) with a flow rate of 15 μL/min. The temperature was kept constant at 60 °C. At the end of the experiment, the effluent was collected in an Eppendorf tube. In addition, 450 μL of methanol were pumped through the microreactor with a flow rate of 150 μL/min in order to collect any amount of substrate or product that might have not been collected. The reaction product was determined as described in Section 2.9.8.

The transesterification reaction was also performed in batch systems, catalyzed by free CALB and its commercial immobilized form (Novozyme 435). The enzyme concentration used for free CALB was 0.03 mg/mL (1.7 μL/mL) and for Novozyme 435 was 0.3 mg/mL (comparable with the amount of protein immobilized in the microreactor system). The lipases were added in 1 mL of 2 mM ethyl ferulate dissolved in 100% *v*/*v* Bet:Gly and incubated for 4 h at 60 °C and 800 rpm. At the end of the experiment, the reaction product was analyzed through HPLC, as described in Section 2.9.8. All measurements were conducted in triplicate.

#### 2.9.8. HPLC Analysis

The transesterification reaction of ethyl ferulate with glycerol was analyzed through high-performance liquid chromatography (HPLC) in an HPLC system equipped with a μBondapak C18 reversed-phase column (particle size 10 μm, length 300 mm, diameter 3.9 mm) and a diode array UV detector. The mobile phase consisted of 40% Acetonitrile (A) and 60% water with 0.1% acetic acid (B) and the elution was isocratic with a flow rate of 1 mL min^−1^. The temperature of the column was set to 35 °C and the injection volume was 20 μL. The substrate ethyl ferulate and the transesterification product glyceryl ferulate were detected at 320 nm. A 100 μL sample of the reaction mixture was obtained at the beginning (0 h) and again at the end of the reaction time (4 h), respectively, and was added to 300 μL of HPLC Water, 20 μL from which were injected into the system for the analysis. No dilution was performed for the methanol effluent. Blank samples were also prepared and analyzed. Calibration curves were constructed by different concentrations of ethyl ferulate. The total conversion yield was determined by the decrease in the amount of ethyl ferulate.

## 3. Results

In the first part of this study, the effect of various DESs on the biocatalytic performance of the immobilized CALB on 3D-printed PLA structures was investigated. Biocatalytic studies were conducted in 3D-printed PLA well plates where the enzyme was immobilized in the interior walls, according to the methodology presented in our previous works [28,34]. The surface of the 3D-printed PLA structures was modified with PEI, which has been previously demonstrated as an effective modification agent for 3D-printed PLA structures [29], while it has also been exploited in enzymatic studies for its protective role against biomolecules and its stabilization effect on immobilized lipases [35,36,37].

### 3.1. Characterization Studies of Modified 3D-Printed PLA Scaffolds

#### 3.1.1. X-ray Photoelectron Spectroscopy (XPS)

**Figure 2:**
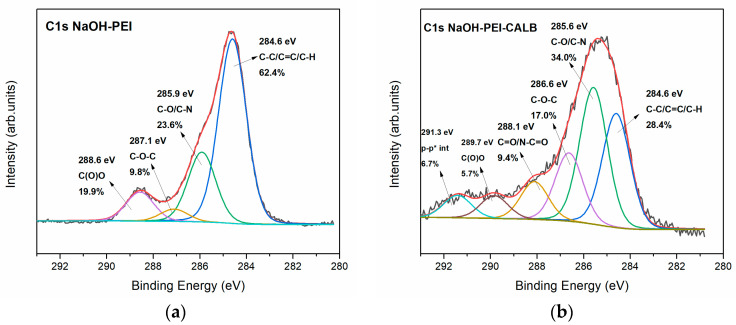
C1s photoelectron spectra for the modified 3D-printed PLA structures (**a**) before enzyme immobilization, (**b**) after enzyme immobilization.

In order to investigate the surface modification of the 3D-printed PLA structures before and after enzyme grafting, XPS analysis was performed. Figure 2 shows the high-resolution C1s photoelectron spectra of (a) NaOH-PEI, and (b) NaOH-PEI-CALB. The fitted peaks reveal the characteristic bonds on the surface of the modified 3D-printed structures before and after the enzyme immobilization. In Figure 2a the four fitted peaks are attributed to C-C/C=C/C-H bonds at 284.6 eV, C-O and C-N bonds at 285.9 eV, C-O-C at 287.1 eV, and carboxylic bonds centered at 288.6 eV. All these functionalities are ascribed to the surface of the reduced surface of PLA after treatment with NaOH, as stated in our previous work [22], and the PEI moieties that have been deposited on the surface. The carbon spectrum after the enzyme immobilization changes significantly, and this is compelling evidence of the successful interaction of the enzyme and the surface. The C-N peak centered at 285.6 eV (Figure 2b) increases due to the amine-rich environment of the enzyme on the surface. A new peak revealed at 288.1 eV provides proof of the covalent immobilization by which the enzyme interacts with the amine groups of the PEI [38]. It is noteworthy that the newly fitted peaks revealed at high energy levels (291.3 eV) may derive from some p-p* electrostatic interactions between adjacent enzymes.

#### 3.1.2. Scanning Electron Microscopy (SEM)

**Figure 3:**
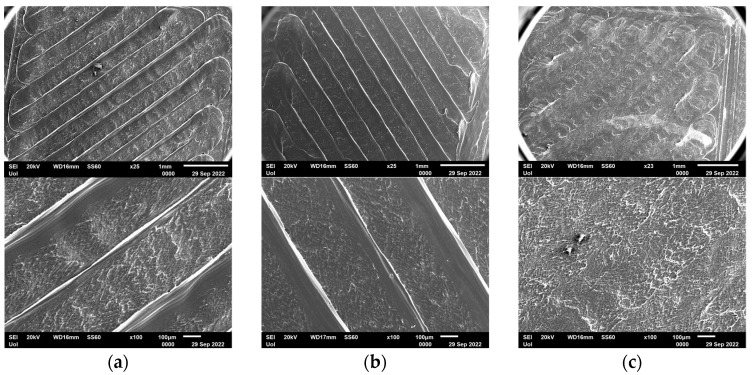
SEM micrographs of modified PLA structures. (**a**) modified PLA, (**b**) modified PLA incubated in 100% DES for 2 h, (**c**) modified PLA incubated in 100% DES for 24 h.

SEM analysis was conducted in order to investigate the effect of a DES on the surface morphology of modified 3D-printed PLA structures. Representative micrographs of the SEM study are presented in Figure 3. Modified PLA structures were incubated at 60 °C in a 100% *v*/*v* Bet:Gly solution for 2 h and for 24 h, which were the most extreme conditions for the intended experimentation in the next stages of this work. The SEM study revealed a slight distortion of the surface of the modified PLA after interaction with the DES solution. The effect was already obvious from 2 h incubation, while after 24 h incubation the distortion was more profound. Micrographs with higher magnification can be found in the supplementary material (Figure S3) where it is determined that the surface quality before and after DES incubation does not change significantly. As a result, the PLA structures maintained their macroscopic rigidity, so they were exploited for further biocatalytic studies to reassure immobilized enzyme performance on these structures.

Previous studies have demonstrated that organic solvents, which are also commonly used in biocatalysis research, cause severe distortion of PLA structures and cannot be used for long exposures times [39,40], so this study shows that DESs can be proposed as alternative solvents for biocatalytic applications without causing severe deformation of the 3D-printed structure.

### 3.2. Enzyme Catalytic Performance in Well plates

#### 3.2.1. Effect of DESs on the Activity of Immobilized CALB

**Figure 4:**
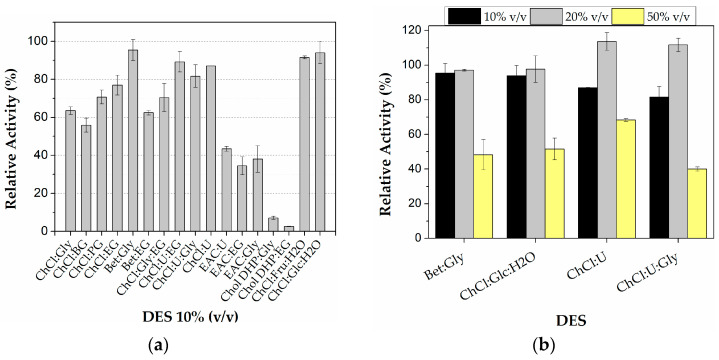
Relative activity of immobilized CALB on PLA well plates (**a**) in various 10% *v*/*v* DES:buffer solutions and (**b**) in increased concentrations of certain ones. 100% is defined as the activity of the immobilized enzyme in buffer (0% *v*/*v* DES). Reaction conditions: 0.25 mM pNPB in 50 mM phosphate buffer pH 7.5, 10, 20, or 50% *v*/*v* DESs, 40 °C, reaction time 5 min.

The activity of the immobilized CALB on PLA multi-well plates was initially screened at various DES-buffer solutions in which the concentration of each DES was 10% *v*/*v*. As can be seen in Figure 4a, the activity of the enzyme depended on the DES used. Specifically, among the DESs whose HBA is ChCl, the best activity was observed in the case of the DESs ChCl:Fru:H_2_O (92%) and ChCl:Glc:H_2_O (94%), and among the DESs that contain Urea as HBD, ChCl:U (87%), ChCl:U:EG (89%), and ChCl:U:Gly (82%). The activity of the immobilized lipase in the aqueous solutions that contain DESs whose HBDs are alcohols was lower (56–77%). Concerning the DESs Bet:Gly and Bet:EG, the enzyme activity was found to be the highest in the presence of Bet: Gly (95%) and was similar to the activity that was observed in the case of the ChCl-based DESs that contain sugars (92% and 94%). Furthermore, the enzyme activity was even lower in the presence of 10% *v*/*v* EAC-based DESs (35–43%) and even more decreased in the presence of Chol DHP-based DESs (3–7%).

Elgharbawy et al., who studied the role of sugar-based NaDESs on the hydrolytic activity of various lipases including CALB (recombinant) immobilized on Immobead 150, also reported that the activity of the lipases in the hydrolysis of pNPP depended on both the nature of NaDESs and the type of lipase used. Specifically, when they tested the aqueous NaDES ChCl:Fru:H_2_O (5:2:5), while it showed a stabilizing effect in most of the lipases, the activity of CALB dropped to 40% compared to the activity in the buffer, whereas in the case of ChCl:Glc:H_2_O (5:2:5), an activation up to 140% was observed [41]. Kim et al., who also studied the effect of several DESs (10% *v*/*v*) on the *Candida rugosa* lipase activity on the pNPP hydrolysis, reported an activation of the lipase in the ChCl-based DESs [42]. Concerning Bet-based DESs, Nian et al. observed an activation of CALB in the hydrolysis of pNPP in Bet:Gly [43], while Ribeiro et al., who used up to 20% m/m of various ChCl- and Betaine hydrochloride-based DES, demonstrated that a stabilization, or even an activation, took place [14]. In the present study, the presence of 10% *v*/*v* EAC-based DESs resulted in diminished hydrolytic activity compared to the results of another work [44]. In the case of the Chol DHP-based DESs, Álvarez et al. studied the activity of CALB in the hydrolysis of p-nitrophenyl laurate in such DESs and found it to be >60% at the concentration of 10% *v*/*v* [45].

From all the above it can be deduced that a certain DES may have a different impact on the enzymatic activity of a lipase depending on the type of the lipase and its form (free or immobilized), as well as on the nature (combination of HBD/HBA) and concentration of the DES used, as the physicochemical properties of the medium varies in terms of polarity, viscosity, density, and pH, which are factors that affect the enzyme structure or/and the enzyme-substrate interaction, and. thus, the catalytic activity, leading to enhanced or lowered yields [46]. However, in the current study, sugar-based, Urea-based, and Betaine-based DESs seem to have the best impact on the activity of immobilized CALB on PLA wells.

In order to further investigate the influence of the concentration of the DESs on the enzymatic activity of the immobilized CALB on the PLA-wells, the DESs Bet:Gly, ChCl:Glc:H_2_O, ChCl:U, and ChCl:U:Gly in which the enzyme demonstrated high activity (in 10% *v*/*v*) were selected and tested in increasing concentrations (20 and 50% *v*/*v*). The results are presented in Figure 4b. For all the DESs examined, it can be deduced that a slight rise in their concentration (20% *v*/*v*) led to enhanced activity (97% for Bet:Gly and 98% for ChCl:Glc:H_2_O) and even to an observed activation of the lipase in the case of the U-based DESs (about 110%), whereas a greater rise in the concentration (50% *v*/*v*) resulted in lower activity (up to 68%). The activation of CALB in these media has also been reported for *Candida rugosa* lipase [42]. The decrease in the enzyme activity noted in higher concentrations of DESs was also observed elsewhere [14,41] and may be associated with the formation of more viscous solutions in which mass transfer phenomena take place and hinder the enzyme-substrate interaction [41].

#### 3.2.2. Effect of DESs on the Stability of Immobilized CALB

**Figure 5:**
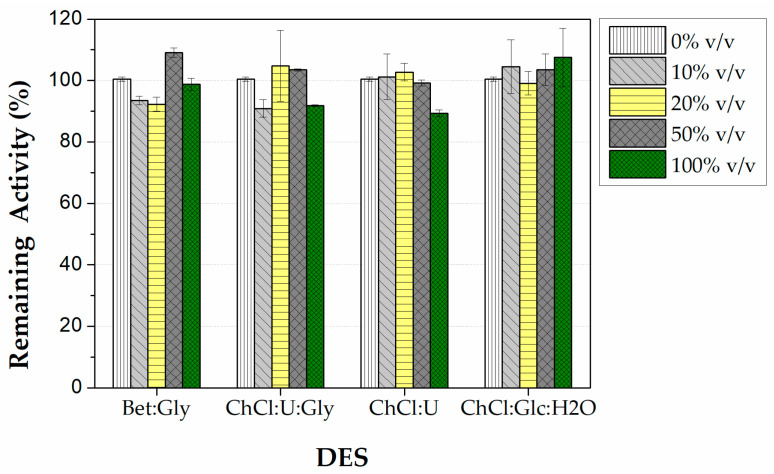
Stability of immobilized CALB on PLA wells after 1h of incubation at different concentrations (0–100% *v*/*v*) of particular DESs at 40 °C. One hundred percent is defined as the initial activity (before the incubation) in buffer (0% *v*/*v* DES). Reaction conditions: 0.25 mM pNPB in 50 mM phosphate buffer pH 7.5, 40 °C, reaction time 5 min.

The stability of the immobilized CALB was examined in buffer (0% *v*/*v* DES) and different concentrations (10, 20, 50, and 100% *v*/*v*) of the four selected DESs. The remaining enzymatic activities after 1h incubation at 40 °C are shown in Figure 5. In general, the remaining activity of the immobilized enzyme was ≥90% in all concentrations of DESs tested, indicating that these selected solvents did not destabilize the immobilized lipase. More specifically, in the low-to-medium concentrations of the U-based DESs (10–50% *v*/*v*), the remaining activity was up to 105%, showing a slight enzyme activation. It is interesting to note that even at 100% *v*/*v* concentration of U-based DESs the immobilized enzyme retained 90% of its initial activity. A significant stabilizing effect was also observed in the case of a high concentration of Bet:Gly, and especially in ChCl:Glc:H_2_O, where no significant loss of activity was observed at any concentration, demonstrating the protective role of these sugar-based DESs. This stabilizing effect of these DES is similar to that observed for other lipase preparations [41,43,47].

#### 3.2.3. Storage Stability of Immobilized CALB on PLA Well plates

**Figure 6:**
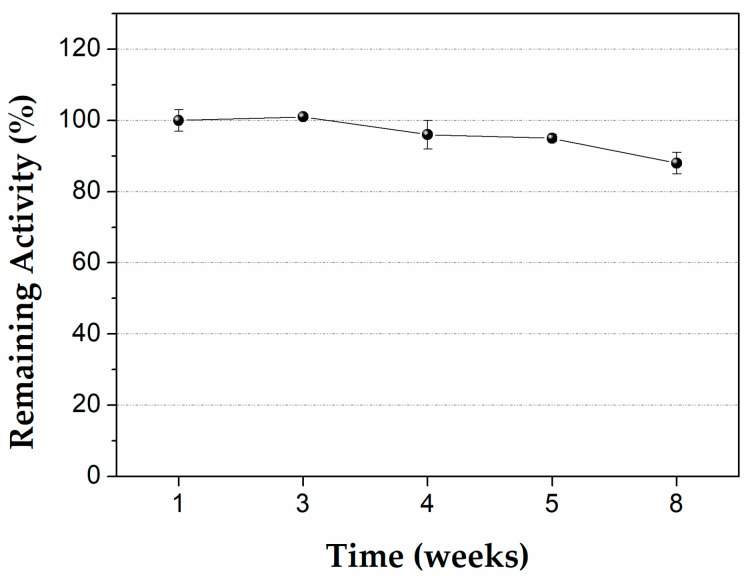
Storage stability of immobilized CALB on PLA wells, at 4 °C. One hundred percent is defined as the initial activity (activity before the storage). Reaction conditions: 0.25 mM pNPB in 50 mM phosphate buffer pH 7.5, 40 °C, reaction time 5 min.

The storage stability of the immobilized lipase on PLA wells was also investigated. The PLA wells with immobilized CALB were stored at 4 °C and the remaining activity of the immobilized enzyme was measured over a period of two months. As can be seen in Figure 6, the biocatalyst retained 90% of its initial activity after a storage time of two months, which is similar to that observed for immobilized CALB on lysine-modified magnetic nanoparticles [48] and remarkably higher than that observed for the immobilized CALB on functionalized mesoporous material MCM-41, where a remaining activity of 50% after incubation for 5.7 weeks at 4 °C was observed [49].

### 3.3. Enzyme Performance in PLA Microreactors

In the present work, 3D-printed PLA continuous-flow microreactors were developed and used for biocatalytic transformations in DES-based media. In this case, the DES Bet:Gly was selected for two reasons; firstly, the lipase activity and stability were among the highest that were presented according to the screening test in PLA wells, and, secondly, this DES can fulfill the 2-in-1 concept for DES elaboration in biocatalysis, acting both as a solvent and a substrate in the transesterification of ethyl ferulate with glycerol.

The developed microreactor operated under a laminar flow regime (Reynolds number < 2100) and the assays were performed using an automated syringe pump, an incubator for reaction temperature control, and Tygon tubing for the intermediate conjunctions, as demonstrated in Figure S1.

#### 3.3.1. Effect of Enzyme Concentration

**Figure 7:**
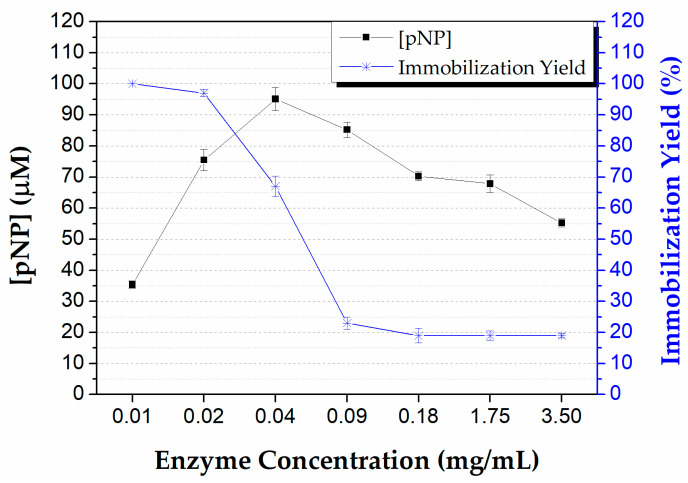
Effect of enzyme concentration on the microreactor’s biocatalytic performance and immobilization yield. Reaction conditions: 0.25 mM pNPB in 50 mM phosphate buffer pH 7.5, 40 °C, flow rate 300 μL/min (*t*_R_ = 30 s).

The preparation of a viable biocatalytic system lies in the determination of the proper amount of enzyme that will be used in the immobilization process, as the cost of such systems strongly depends on this. In order to determine the optimal concentration of CALB for its immobilization in the microreactor, several concentrations were used and associated with the amount of pNP produced by each biocatalytic system. In addition, the immobilization yield was estimated, by measuring the activity of the enzyme solution before and after the microreactor loading. As shown in Figure 7, the increase in enzyme concentration was followed by an increment in the product formation until a critical point (0.04 mg/mL), beyond which the productivity of the biocatalytic system decreased gradually. In terms of the immobilization yield, almost all the CALB seemed to have been immobilized up to a concentration of 0.02 mg/mL. At higher enzyme amounts, the immobilization yield sharply declined, indicating that the immobilization capacity of the reactor was limited. At the concentration where maximum enzyme productivity was observed (0.04 mg/mL), the immobilization yield was 70%. The decline in enzyme productivity possibly occurred due to an oversaturation of enzyme molecules in the microreactor that led to inactivation or hindrance. Similar assumptions have been made in previous studies for enzymatic microreactors either with immobilized or free enzyme forms in the interior of microreactors, using different enzymes [22,50,51]. Hence, the optimum enzyme concentration of 0.04 mg/mL was selected for further studies.

#### 3.3.2. Operational Stability in the Presence or Absence of DES

**Figure 8:**
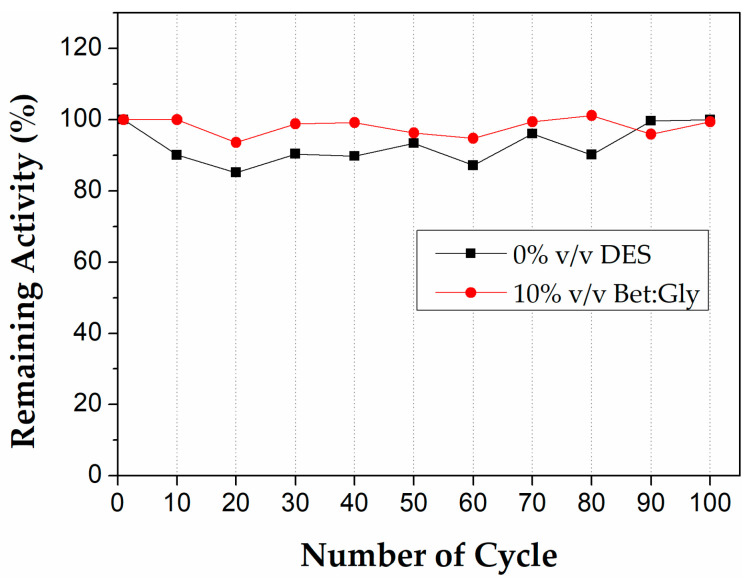
Operational stability of immobilized CALB in PLA microreactors in the absence and the presence of 10% *v*/*v* Bet:Gly for 100 cycles of use. Reaction conditions: 0.25 mM pNPB in 50 mM phosphate buffer pH 7.5, 0 or 10% *v*/*v* Bet:Gly, 40 °C, flow rate 300 μL/min (*t*_R_ = 30 s).

The biocatalytic microreactor system was evaluated in terms of its operational stability by performing 100 successive cycles of use, in the absence or the presence of 10% *v*/*v* Bet:Gly. The hydrolysis of pNPB was used as the model reaction and each cycle was deemed when the whole microreactor volume (150 μL) passed through the microreactor. As presented in Figure 8, the immobilized CALB in PLA microreactor retained its initial activity at 100% after 55 min of continuous use in both 0% and 10% *v*/*v* Bet:Gly. This suggests that the presence of DES did not affect the operational stability of the biocatalyst, thus rendering its use feasible in long-lasting hydrolysis reactions. The remarkable operational stability of immobilized enzymes in microreactor systems has been previously reported both for hydrolytic and synthetic bioprocesses, suggesting the potential of microfluidic systems in terms of process efficiency and sustainability [22,50,52].

#### 3.3.3. Thermal Stability of Immobilized CALB in Microreactor

**Figure 9:**
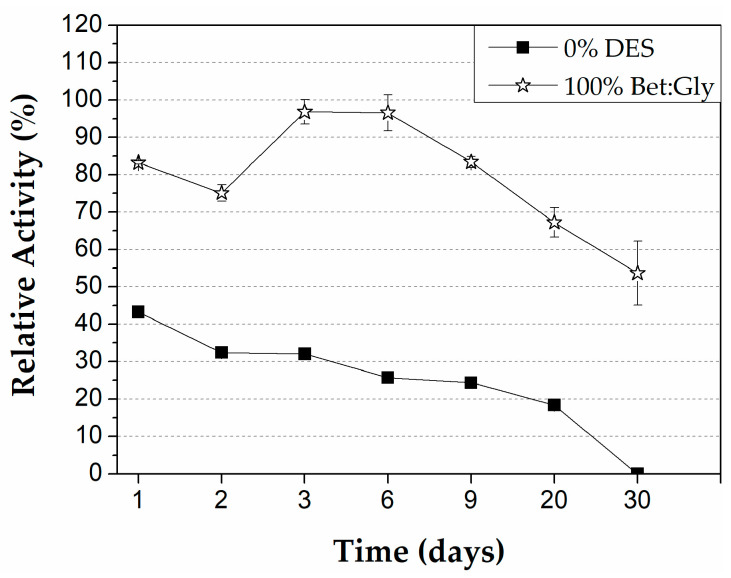
Thermal stability of immobilized CALB in PLA microreactor, at 60 °C, after incubation in 100% DES Bet:Gly and 100% buffer. Reaction conditions: 0.25 mM pNPB in 50 mM phosphate buffer pH 7.5, 40 °C, flow rate 300 μL/min (*t*_R_ = 30 s).

The robustness of the enzymatically modified PLA microreactor was also tested by filling the microreactor with 100% Bet:Gly and incubating at 60 °C. In this manner, the stability of the immobilized lipase would be evaluated, and insight would be gained into the capability of the microbioreactor to be used in synthetic reactions that include this DES. So, at specific points in time, the DES was removed, the microbioreactor was washed and dried and the activity of the lipase was measured. The results regarding the stability of the immobilized CALB in the PLA microreactor for a period of one month are presented in Figure 9. In the case of the incubation in DES, the lipase demonstrated great stability, as it retained more than 50% of its initial activity after one month compared to the control, where the microreactor was fulfilled with buffer and the activity of CALB was diminished. These results give prominence to the stabilizing effect that betaine-based DES has on the protein molecules. The stabilizing effect of Bet:Gly on free CALB has also been reported elsewhere [43]. A possible explanation for the enzyme stabilization that some DESs offer at elevated temperatures could be that the hydrogen bonding of the DES’s ingredients helps to maintain the enzymatic structure by absorbing the heat of the medium and thus, the enzyme molecules do not get dehydrated [41].

#### 3.3.4. Continuous Flow Kinetics of Immobilized Lipase in the Presence or Absence of DES

To give better insight into the biocatalytic behavior of the immobilized lipase, the effect of the flow rate on the hydrolysis of the model substrate pNPB in the presence or absence of 10% *v*/*v* of the selected DES Bet:Gly was evaluated. According to previous works, mass transfer phenomena are expected to affect the kinetic behavior of enzymes confined in the interior of microreactor systems [53,54]. The flow kinetics and the presence of any diffusion or mass transfer effects that may conceal the true kinetics (Q = 0) were estimated by applying the Lilly–Hornby model, as an adaptation of the classical Michaelis–Menten kinetic model, used for investigating flow kinetics of enzymes immobilized in microreactor systems [55].

**Figure 10:**
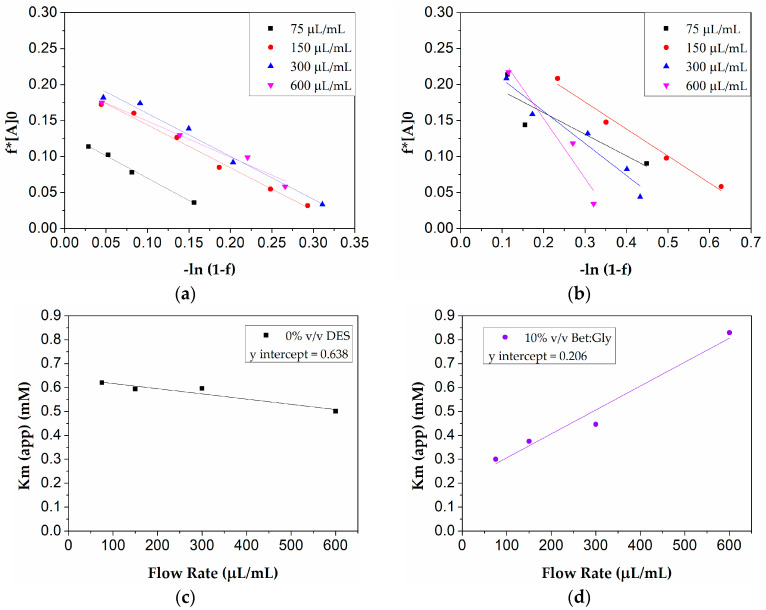
Effect of flow rate on the hydrolysis of p-NPB in CALB immobilized microreactors under continuous flow conditions (enzyme concentration 43 μg/mL, T = 40 °C, pH = 7.5). (**a**) Application of the Lilly–Hornby model on the data collected for different enzyme concentrations and flow rates in a buffer system (0% *v*/*v* DES), and (**b**) in 10% Bet:Gly system. (**c**) Linear fitting of the ***K***_*m*(*app*)_ values against flow rate in a buffer system, and (**d**) in 10% Bet:Gly system.

From the kinetic data collected at an initial substrate concentration of from 0.25 mM to 4 mM, at varying flow rates of from 75 μL/min to 600 μL/min, linear plots of $f{\left[A\right]}_{0}$ versus −ln(1−f) were obtained (Figure 10a,b). The slopes of the lines, that correspond to the ***K***_*m*(*app*)_ values (Table 3), seem to decrease with increasing flow rate, indicating a better performance of the immobilized enzyme in faster residence times. Moreover, the system does not seem to get significantly affected by mass transfer phenomena since the zero flow rate value (0.638 mM) (y-intercept, Figure 10a) does not match the Michaelis constant for the free enzyme form (0.348 mM) [55]. It seems that the accessibility of the substrate to the enzyme molecules may have decreased due to the immobilization process (higher ***K***_*m*(*app*)_ values than the free enzyme) but is not affected by diffusion limitations [53].

However, when 10% *v*/*v* Bet:Gly is added to the system, its kinetic behavior alters significantly. In this case, an increase in the slopes of the lines (Figure 10b,d) is observed with increasing flow rate indicating mass transfer effects. However, moving from the lowest to the highest flow rates tested, only a 2.6-fold increase in the ***K***_*m*(*app*)_ values are observed, proving that the mass transfer effects are not considered strong [53,55]. The ***K***_*m*(*app*)_ for zero flow rate in this case (0.206 mM) is lower than that observed for the free enzyme form (0.392 mM), indicating more effective substrate accessibility in the immobilized enzyme acting in a DES-based system. Such an observation has been previously reported for immobilized enzyme microreactors and is indicative of the increased stabilization of the biocatalyst along with the more effective substrate diffusion within the system [50,55].

Inferring from the above, the designed microreactor system is not remarkably affected by mass transfer phenomena, while the addition of Bet:Gly solvent in the system greatly improves the substrate diffusion within the immobilized enzyme molecules.

### 3.4. Implementation of the Enzyme Microreactor in a Transesterification Reaction

**Figure 11:**
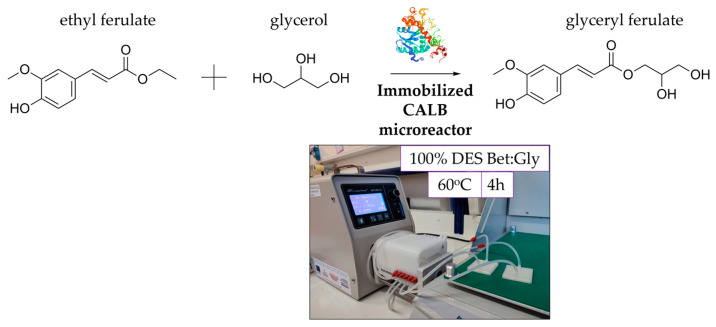
Reaction scheme for the transesterification of ethyl ferulate with glycerol in 100% Bet:Gly DES acting both as solvent and substrate, towards the production of glyceryl ferulate. The reaction was performed in a microreactor system with immobilized CALB, at 60 °C for a total retention time of 4 h (24 runs × 10 min).

Glyceryl ferulate (GF) is a natural compound widely distributed among the plant kingdom that is especially known for its antioxidant and UV light-absorbing properties [56]. However, its isolation from natural sources is limited, so the de novo synthesis methods through esterification and transesterification reactions have been proposed [57,58]. Herein, the transesterification reaction of ethyl ferulate with glycerol towards the synthesis of GF (Figure 11) is demonstrated for the first time in a continuous flow microreactor system, elaborating Bet:Gly as a novel, green reaction solvent, that could play a double role as a solvent component and a reaction substrate at the same time (the 2-in-1 concept). The transesterification reaction was also performed in batch reaction systems. Free CALB and commercially immobilized CALB (Novozym 435) were used under the same reaction conditions and the productivity of the different systems is presented in Table 4.

The HPLC analysis revealed only one reaction product, with a retention time of 3.3 min, which presumably is GF, according to previously published works [57]. Further studies, which are beyond the scope of the present work, would shed light on the identity of this product. Interestingly, the microreactor system presented the highest productivity, which was 23 times higher than that of the commercial immobilized lipase and 1.2 times higher than the productivity of free CALB. Previously published works have showcased similar results [18,50,52,59], confirming the superiority of enzyme microreactors as an alternative to batch reactor systems when productivity is of critical importance.

## 4. Conclusions

The incorporation of novel technologies and solvent systems into biocatalysis is urgent for new reactions to be feasible and enzymes to be more resistant and productive. Herein, 3D printing technology was exploited for the development of carriers for lipase immobilization, in the form of microwell plates and in the form of microfluidic reactors. The 3D-printed PLA structures were properly modified in order to host covalently immobilized lipase, and several biocatalytic assays were performed regarding the effect of DESs on the enzyme performance. Different DESs were shown to have a stabilizing effect on enzymatic activity, even at 100% *v*/*v* DES concentration. The residual activity of the immobilized enzyme at 4 °C was 90% after two months of storage, indicating good storage stability. In 3D-printed microfluidic reactors, a kinetic study showed that the presence of the DES Bet:Gly in the reaction system improved greatly the apparent kinetic constant of the enzyme, while the system was not severely affected by diffusion limitations. It is also noteworthy that the enzyme microreactor system showed exceptional thermal stability after incubation in 100% *v*/*v* DES at 60 °C, retaining 53% of its activity after 30 days of storage, while the microreactor stored in buffer solution had been completely deactivated. These experiments confirmed that the slight surface distortion after DES incubation observed in the SEM study, does not affect the biocatalytic performance of the system; thus making these solvents ideal alternatives for non-aqueous biocatalytic processes. A continuous flow transesterification reaction was also demonstrated in this study, showing the potential of the system to reach productivities significantly higher than batch reaction systems under the same conditions.

This study featured an effective and robust biocatalytic system with immobilized lipase that can be used both in hydrolytic and synthetic processes with the incorporation of environmentally friendly solvents such as DESs. Further optimization, either with different solvents or with different microreactor systems, could upgrade its performance. This microreactor system is based on natural and recyclable starting materials and it is readily reusable, providing a sustainable option for biocatalysts design.

## Figures and Tables

**Table 1:** Printing parameters set by the slicing software.

Parameter	Value
Layer Height	0.16 mm
Infill Density	100%
Printing Temperature	200 °C
Build Plate Temperature	50 °C
Print Speed	80 mm/s
Nozzle Size	0.4 mm
Filament Diameter	1.75 mm

**Table 2:** The prepared DESs used in this study.

DES [HBA: HBD (: HBD)]	Molar Ratio	Name Code
Choline Chloride: Glycerol	1:2	ChCl:Gly
Choline Chloride: Butylene Glycol	1:4	ChCl:BG
Choline Chloride: Propylene Glycol	1:2	ChCl:PG
Choline Chloride: Ethylene Glycol	1:2	ChCl:EG
Betaine: Glycerol	1:3	Bet:Gly
Betaine: Ethylene Glycol	1:3	Bet:EG
Choline Chloride: Glycerol: Ethylene Glycol	1:1:1	ChCl:Gly:EG
Choline Chloride: Urea: Ethylene Glycol	1:1:1	ChCl:U:EG
Choline Chloride: U: Glycerol	1:1:1	ChCl:U:Gly
Choline Chloride: Urea	1:2	ChCl:U
Ethylammonium Chloride: Urea	1:1.5	EAC:U
Ethylammonium Chloride: Ethylene Glycol	1:1.5	EAC:EG
Ethylammonium Chloride: Glycerol	1:1.5	EAC:Gly
Choline Dihydrogen Phosphate: Glycerol	1:3	Chol DHP:Gly
Choline Dihydrogen Phosphate: Ethylene Glycol	1:3	Chol DHP:EG
Choline Chloride: Fructose: H_2_O	5:2:5	ChCl:Fru:H_2_O
Choline Chloride: Glucose: H_2_O	5:2:5	ChCl:Glc:H_2_O

**Table 3:** Apparent Michaelis constant (***K***_*m*(*app*)_) calculated from the Lilly−Hornby model for the microreactor system and Michaelis constant for the free enzyme calculated from the Michaelis−Menten model, under the same reaction conditions.

	Flow Rate (μL min^−1^)	*K*_*m*(*app*)_ (mM)	
		**0% *v*/*v* DES (*R*^2^)**	**10% *v*/*v* Bet:Gly (*R*^2^)**
Free CALB	-	0.35 (0.88)	0.39 (0.93)
Microreactor immobilized CALB	75	0.62 (0.99)	0.30 (0.78)
	150	0.59 (0.98)	0.38 (0.98)
	300	0.60 (0.99)	0.45 (0.95)
	600	0.50 (0.98)	0.83 (0.94)

**Table 4:** Comparative data for the transesterification of ethyl ferulate with glycerol in microreactor and batch systems.

Biocatalytic System	Amount of Enzyme (μg)	Reaction Volume (mL)	Amount of Product (μg)	Reaction Time (h)	Productivity (μg Product min^−1^ μg enzyme^−1^)
Microbioreactor	4.5	0.15	18.6	24 runs × 10 min	17.20 × 10^−3^
Immobilized CALB (Novozyme 435)	30	1	5.4	4	0.75 × 10^−3^
Free CALB	30	1	97.9	4	13.60 × 10^−3^

## Data Availability

Not applicable.

## Supplementary Materials

The following supporting information can be downloaded at: https://www.mdpi.com/article/10.3390/mi13111954/s1, Figure S1: Microfluidic apparatus used in this study; Figure S2: Activity of immobilized CALB on PLA well plates modified with PEI.
