# Supplementary material for: Development of 3D Printed Enzymatic Microreactors for Lipase-Catalyzed Reactions in Deep Eutectic Solvent-Based Media
Source: Micromachines (Basel). 2022 Nov 11;13(11):1954. doi: 10.3390/mi13111954 (PMC9693471; doi:10.3390/mi13111954)
Supplement: Supplementary file 1 [file micromachines-13-01954-s001.zip › micromachines-2017219-supplementary.pdf]

Development of 3D Printed Enzymatic Microreactors for Lipase-Catalyzed Reactions in Deep Eutectic Solvent-Based Media

Myrto G. Bellou [1], Elena Gkantzou [1,*], Anastasia Skonta [1], Dimitrios Moschovas [2], Konstantinos Spyrou [2], Apostolos Avgeropoulos [2], Dimitrios Gournis [2] and Haralambos Stamatis [1,*]

[1] Laboratory of Biotechnology, Department of Biological Applications and Technologies, University of Ioannina, 45110 Ioannina, Greece
[2] Department of Materials Science and Engineering, University of Ioannina, 45110 Ioannina, Greece
* Correspondence: e.gkantzou@uoi.gr (E.G.); hstamati@uoi.gr (H.S.)

SUPPLEMENTARY MATERIAL

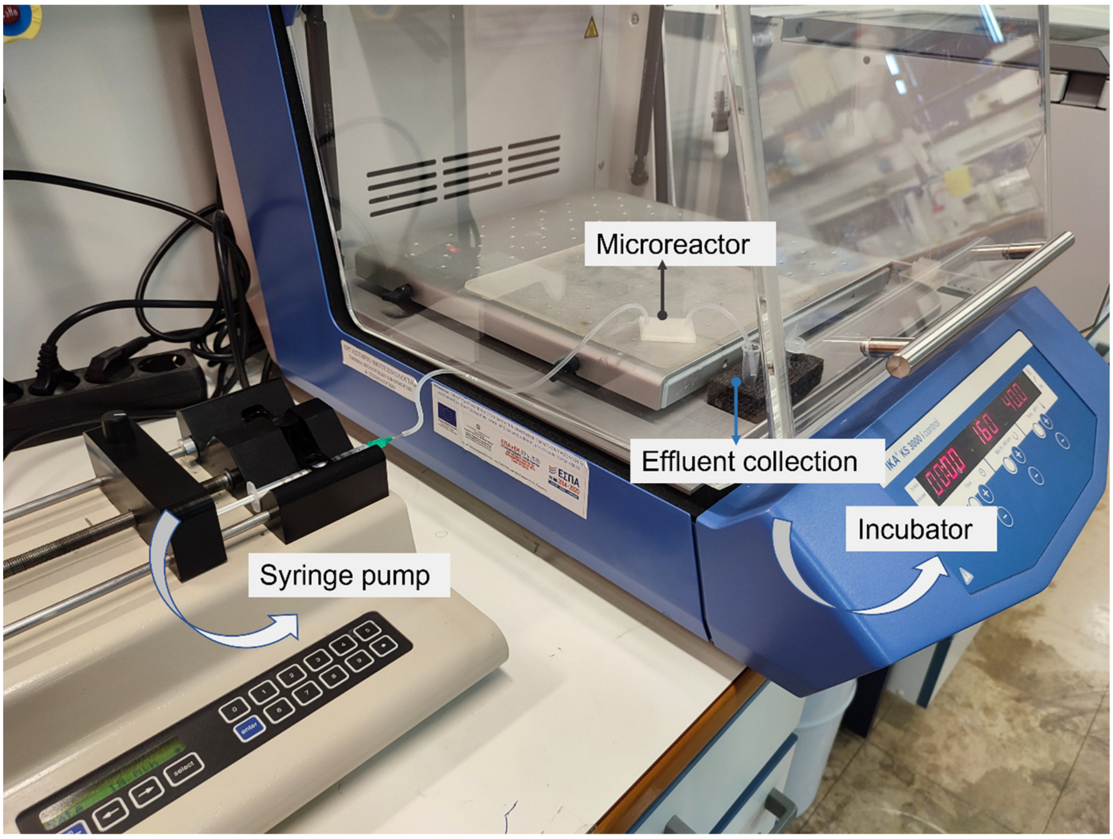

Figure S1. Microfluidic apparatus used in this study. A syringe pump is used to regulate the flow rate of the system. The microreactor is placed inside an incubator to keep the temperature of the system constant, and the effluent of the microreactor is collected in an Eppendorf tube for use in further measurements.

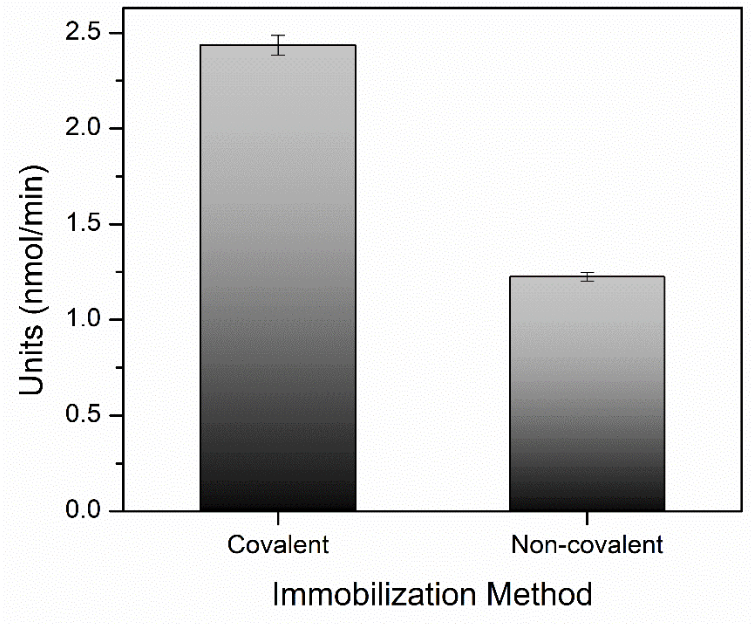

Figure S2. Activity of immobilized CALB on PLA well plates modified with PEI. Covalent refers to the use of a glutaraldehyde as cross-linking agent and non-covalent refers to absorption of the enzyme on the PEI modified surface of PLA. Measurement conditions: 0.25 mM pNPB in 50 mM phosphate buffer pH 7.5, 40 °C, reaction time 5 min.
